# Cell Type Annotation Model Selection: General-Purpose vs. Pattern-Aware Feature Gene Selection in Single-Cell RNA-Seq Data †

**DOI:** 10.3390/genes14030596

**Published:** 2023-02-26

**Authors:** Akram Vasighizaker, Yash Trivedi, Luis Rueda

**Affiliations:** School of Computer Science, University of Windsor, Windsor, ON N9B 3P4, Canada

**Keywords:** cell type annotation, scRNA-seq data, gradient boosting, domain-specific features, feature selection

## Abstract

With the advances in high-throughput sequencing technology, an increasing amount of research in revealing heterogeneity among cells has been widely performed. Differences between individual cells’ functionality are determined based on the differences in the gene expression profiles. Although the observations indicate a great performance of clustering methods, manual annotation of the clusters of cells is a challenge yet to be addressed more scalable and faster. On the other hand, due to the lack of enough labelled datasets, just a few supervised techniques have been used in cell type identification, and they obtained more robust results compared to clustering methods. A recent study showed that a complementary step of feature selection helped support vector machine (SVM) to outperform other classifiers in different scenarios. In this article, we compare and evaluate the performance of two state-of-the-art supervised methods, XGBoost and SVM, with information gain as a feature selection method. The results of the experiments on three standard scRNA-seq datasets indicate that XGBoost automatically annotates cell types in a simpler and more scalable framework. Additionally, it sheds light on the potential use of boosting tree approaches combined with deep neural networks to capture underlying information of single-cell RNA-Seq data more effectively. It can be used to identify marker genes and other applications in biological studies.

## 1. Introduction

In living organisms, there are a great variety of cells that can be distinguished with the help of single-cell RNA sequencing (sc-RNA sequencing) technology. Single-cell RNA sequencing (scRNA-seq) is a novel sequencing technology that involves individual cell information and can be used in cell heterogeneity studies. Studying different types of cancer, detecting unknown tumours and tumour heterogeneity, drug discovery, diagnosis, and prognosis are a few numbers of the new opportunities for research in this scope.

Identifying cell type heterogeneity is one of the first fundamental steps in an in-depth analysis of single-cell RNA sequencing data. Hidden diversity and characteristics of a particular cell type can be found via deferentially expressed genes (DEGs). Machine learning approaches can be effectively used to identify hidden differentiation in the expression profiles of the genes with high probability. scRNA-seq data comes with a variety of limitations. The highlighted one is the lack of annotation for most of the data which are publicly available. In a general single-cell RNA-seq downstream analysis, clustering techniques are widely used to reveal groups of cells and cell types. However, setting up the parameters, including the number of clusters, is a challenging point [1]. For instance, several methods are compared in [2]. Among them, SC3 [3], CIDR [4], Ascend [5], SAFE-clustering [6], TSCAN [7], and [8] all possess built-in methods for estimating the optimal number of clusters. Although Ascend and CIDR underestimated the number of clusters, SC3 and TSCAN tend to overestimate. In addition, the group of cells identified by the clustering methods requires an additional annotation step with the corresponding cell types using canonical marker genes and reference databases. Hence, the conventional workflow based on clustering and marker genes is not scalable due to manual annotation. The lack of ground-truth information and tool benchmarking makes it more complex to evaluate the model. Therefore, manually annotating the output is a time-consuming and non-reproducible procedure in clustering methods. The other limitation of scRNA-seq data is caused by biological effects during sequencing. This leads to a zero-inflated read counts matrix with thousands of zeros in expression values, which may mislead downstream analyses. Cell types are often distinguished by calculating the differentiation of expression levels of only the most informative genes. Hence, finding known marker genes among thousands of genes with almost zero information is essential in scRNA-seq data analyses. This is either called dimensionality reduction or feature selection and affects the final result directly. Although the current unsupervised methods show superiority in the performance when combined with feature selection methods, the biological significance of the results is still important for the understanding of the underlying biological information and requires further manual gene set enrichment analysis [1,9]. Since feature selection plays a significant role in domain-dependent problems, a wide range of supervised techniques shows superiority in the performance utilizing feature selection methods. Supervised techniques have increasingly developed for the automatic identification and annotation of cell types. Moreover, using annotated data, we can evaluate and compare the model by systematically estimating the performance metrics. A comparative study in [10] reviewed 22 supervised techniques, including random forest (RF), which is based on decision tree rules. To assess the probability of a correct label, decision trees inherently select informative features and estimate the minimum number of features needed to create a model. Among the other choices in decision tree categories, XGBoost shows its capability in all scenarios [11]. According to this, CaSTLe [12] was proposed based on an XGBoost model under transfer learning workflow and showed satisfactory classification accuracy compared to two linear models. The idea behind CaSTLE is to use a robust univariate feature engineering workflow followed by the application of a pre-tunedXGBoost model. In the feature selection workflow first, genes with the top mean level of expressions and mutual information were selected, and correlated genes were removed; then, considering pre-defined ranges, genes were categorized. Transfer learning uses information from one scRNA-seq dataset to annotate another one.

Additionally, the ensemble learning schema combines weak learners’ voting for an accurate final vote on similarity search space. For instance, EDGE [13] has utilized this approach on simulated data and learned an ensemble version of similarity matrices into a single embedding space of data, as well as optimizing through stochastic gradient descent. In EDGE, dimensionality reduction and feature gene extraction were used in an ensemble approach in such a way that the problem of finding similarity among features, was broken down into small weak learners. The final similarity matrix achieved shows a common similarity space among all learners. SMaSH [14], on the other hand, is designed explicitly for gene ranking and calculating the significance score of marker genes from scRNA-seq data. Focuses on marker genes ranking, SMaSH compares tree-based and neural network-based approaches. It uses predefined cell types (labels) to categorize cell-specific genes before feeding them into several weak classifiers. In a benchmarking experiment, SMaSH compared the ensemble mode with the network mode. Its performance was evaluated using tree-based models, including XGBoost. Compared to the other two ensemble models and deep neural networks, XGBoost shows excellent performance in most scenarios. Although according to the observations in [15], XGboost failed to detect small changes in expression levels and consequently distinguish cell sub-types. It is a much faster and simpler approach compared to the neural network model. XGBoost is well-suited to large datasets by performing in parallel. Moreover, in our recent comparative study, it has been shown that the support vector machine (SVM), with the help of information gain (IG), as a feature selection method, outperformed the other approaches [16]. The study was performed on nine different experiments composed of three different state-of-the-art popular classifiers combined with three general-purpose feature selection methods. Classifiers, including random forest, *K*-NN, and SVM and feature selection methods, including Analysis of Variance (ANOVA) F-value, Information Gain, and Chi-squared considered complementary of the classifiers. One of the challenges covered in this study was selecting cell-specific genes in the feature selection step. A benchmark study was performed based on the number of selected features. However, it remains for a exploration of general-purpose feature engineering techniques against domain-specific ones, and in particular pattern-aware techniques to be done. When reviewing different approaches, including supervised and unsupervised, for cell type annotation based on scRNA-seq data, there is no comparison with the XGBoost method. Precisely, the power of XGBoost and SVM was proven in the previous studies and XGBoost performs in a faster and simpler way. Moreover, SVM together with a general-purpose feature selection had been shown as a high-performance method in the supervised cell-type annotation. In this study, inspired by the recent works completed in cell-type classification, we compared two forefront approaches; the general-purpose model, a SVM classifier with information gain feature selection method and XGBoost tree with its inherent feature selection strategy. This paper guides users and practitioners to select the most proper model based on the inherent features of their datasets.

## 2. Materials and Methods

### 2.1. Framework

**Figure 1:**
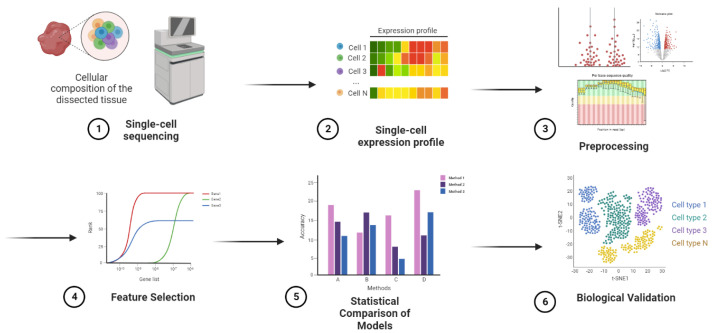
Pipeline overview of the experiments.

The schematic view of Figure 1 depicts the pipeline in a cell-type annotation process. First, the raw read count matrix is generated using high-throughput sequencing technologies (Figure 1, step 1). These raw data includes expression profiles of thousands of cells separately (Figure 1, step 2). Performing pre-processing, including filtering, normalization, and scaling, gives us ready-to-use data for the computational step (Figure 1, step 3). Then, the most informative features are extracted in the feature selection (Figure 1, step 4) to be used by classification models. Finally, cell types are predicted and annotated by the method with higher accuracy (Figure 1, step 5). As a demonstration of the high performance, a gene set enrichment analysis on the selected features was performed, and the results highlight the power of the model in annotating cell types (Figure 1, step 6). The last step is not necessary for supervised approaches. However, it could play a verification phase in the biological context.

To validate our models, we considered the most commonly used evaluation metrics, namely accuracy, precision, and recall, to systematically estimate and compare the performance of our models. To this end, we used 10-fold cross-validation to test and train the model. Additionally, we tuned XGBoost parameters as follows: (1) the regularization parameter value to create a new split in trees, gamma is set to 0.2, 0.1, and 0 for Data1 to Data3, respectively. (2) ($Max_{d}epth$ and $min_{c}hild_{w}eight$) of the tree, which typically control overfitting, were fine-tuned to (10, 3), (5, 3), and (10, 1) for Data1 to Data3, respectively. (3) $colsample_{b}ytree$, which determines what portion of features will be used, was set to 0.5, 0.4, and 0.3 for Data1 to Data3, respectively.

We used Scikit-learn [17] in Python version 3.7 to perform computational algorithms, and GSEA [18] for biological validation.

### 2.2. Dataset

To evaluate the performance of the model, we used public, annotated scRNA-seq datasets with accession numbers GSM2230757, GSM2230758, and GSM2230762 under series GSE84133 [19] extracted from NCBI’s Gene Expression Omnibus [20]. These datasets include transcripts of pancreatic from human and mouse donors. Pancreatic cells are divided into 14 groups of previously characterized cell types, mainly including alpha, beta, acinar, delta, quiescent, activated pancreatic stellate, endothelial, and ductal cells. The existence of these cell types is validated with immuno-histochemistry stains [19] so that it can be a good resource for discovering cell types. The details of the datasets used in this study are listed in Table 1.

### 2.3. Data Pre-Processing

Raw read count matrices contain low-quality RNA sequencing information based on differential expression levels. Data pre-processing is performed to ensure removing any weakly expressed genes or low-quality cells, including damaged, dead, or degraded during sequencing, and are represented by a low number of expressed genes in the read count matrices. We followed the standard pre-processing pipeline in scRNA-seq data analysis [21]. Based on this pipeline, cells with less than 200 expressed genes and genes expressed in less than three cells are filtered out. In Data1, for example, we first filtered out 5387 low-expression genes that were detected in less than three cells and kept 14,739 genes. Further analysis of the data distribution showed low-quality cells and led to removing seven cells. After per-gene quantification, we selected a subset of highly variable genes to use in downstream analyses. To this end, we defined the set of highly variable genes given a normalized dispersion higher than 0.5 after normalization and obtained 2546 genes at the end. We used Scanpy [22], a specifically designed package to work with scRNA-seq datasets, for pre-processing steps.

### 2.4. Hyperparameter Tuning

Hyperparameter tuning, also known as hyperparameter optimization or model selection, is the process of systematically searching for the best combination of hyperparameters to optimize the performance of a machine learning model. Hyperparameters are parameters that are not learned from data but are set before training. Examples of hyperparameters for XGBoost include the learning rate, max depth, gamma, minimum child weight, and column sample by the tree. In this research, the process of tuning these parameters has been completed automatically using Bayesian optimization. Bayesian optimization is a method for efficiently searching for the best set of hyperparameters of a model. The basic idea is to use a probabilistic model, such as a Gaussian process, to model the function that maps from hyperparameters to the performance of the model on a given task. Mathematically, Bayesian optimization can be formulated as an optimization problem in which we want to find a set of hyperparameters that maximize the expected performance of the model, given the current state of the probabilistic model. The expected performance is given by the mean of the model, and the uncertainty in the model is represented by the variance. The acquisition function, such as the expected improvement, is used to balance the exploration and exploitation of the search space. To optimize the acquisition function, we optimize the hyperparameters of the probabilistic model to maximize the expected improvement. This is done by using gradient-based optimization algorithms, such as L-BFGS, or using more sophisticated methods, such as Hamiltonian Monte Carlo. In summary, Bayesian Optimization is a powerful method for tuning a machine learning model’s hyperparameters by using a probabilistic model to guide the search for the best set of hyperparameters and balancing exploration and exploitation using an acquisition function.

### 2.5. Feature Selection

Feature selection is a non-separable part of any algorithms that work with large-scale data due to the curse of dimensionality. The existing thousand genes expressed in each individual cell in the scRNA-seq dataset make it high-dimensional, which required a reduction in the number of genes. The idea behind gene selection in cell type identification is motivated by the fact that cell types are often distinguished by only a few essential genes known as biomarkers. The effectiveness of three general-purpose feature selection methods was explored in cell-type classification problems in [16], including Analysis of Variance (ANOVA) F-value, Chi-squared, and information gain. The findings show that information gain yields the best biomarkers among all other models. Information gain is defined based on impurity and entropy. The group with the higher information gain possesses less uncertainty. The importance of a feature is estimated by considering the information gained from each feature. It is defined as the difference between before and after considering feature *X* in the classification process, as shown in Equation (1) [23].

(1) $$IG\left(X\right)=\sum_{i}U\left(P\left(C_{i}\right)\right)-E\left[\sum_{i}U\left(P\left(C_{i}|X\right)\right)\right]$$

where IG(X) represents the information gain from feature *X*. *U* represents uncertainty function, $P(C_{i})$ represents the probability of class $C_{i}$ before considering feature *X*, and $P(C_{i}|X)$ represents the posterior probability of class $C_{i}$ after considering feature *X*.

On the other hand, the feature selection algorithm in the XGBoost considers sparsity in the data and defines a default direction for missing values. Hence, it simplifies the classification process by utilizing inherent sparsity patterns in the data. Therefore, it divides data into two supergroup samples with missing and present values. XGBoost exploits the sparsity to make the computational complexity linear proportional to the number of existing values in the input matrix [11].

### 2.6. XGBoost

Extreme Gradient Boosting, XGBoost, is a scalable and widely-used decision tree gradient-boosted algorithm that offers state-of-the-art results on many machine learning problems. It provides a statistical model that captures the dependency of large datasets considering the sparsity of the data and has been shown in a wide range of standard classification applications [24]. XGBoost is reported as the top first-ranked method among the most popular ones outperforming the other popular solutions. The second-ranked method, deep neural nets, also obtains better results when combined with XGBoost [11]. Similar to the random forest, a Gradient boosting decision tree follows an ensemble learning algorithm and is under a gradient tree-boosting framework. Ensemble learning algorithms combine multiple models to obtain an average of all models.

The idea follows from the existing Gradient boosting algorithms with minor improvements in the regularized objective. Unlike decision trees, regression trees include a continuous weight on each leaf. For a given data, the regression tree uses the decision rules to classify it into different groups in the leaves. It calculates the overall prediction score by summing up the score weights in the leaves. The regularization objective function has to be minimized as follows:

(2) $$\iota \left(\phi \right)=\sum_{i}l\left({\hat{y}}_{i},y_{i}\right)+\sum_{k}\Omega \left(f_{k}\right)$$

$$where\;\Omega \left(f\right)=\gamma T+\frac{1}{2}\lambda \omega^{2}$$

Here, ι is the loss function to calculate the difference between the predicted and actual class, ${\hat{y}}_{i}$ and $y_{i}$, respectively. The term Ω controls the complexity of the model, i.e., the regression tree functions. The term γ helps avoid over-fitting utilizing the final weights. The regularized greedy forest (RGF) model [25] uses a similar regularization method, but it is more complex. Parallelization is another positive point of XGBoost.

## 3. Results and Discussion

The first objective of this work was to evaluate the accuracy of the main classifiers (i.e., SVM, *k*NN, and RF) with a group of genes extracted from a pioneer general-purpose feature selection method, information gain. The second scenario was defined with genes obtained using the inherent approach in the XGBoost tree, which uses the latent pattern in scRNA-seq data. We calculated the average of all measurements when comparing the results. The number of features is determined based on the one with the highest final predictive accuracy. Results are presented in Table 2, Table A1 and Table A2. Overall, our findings indicate that XGboost obtained the first rank among other methods in terms of accuracy and recall. On the other hand, when looking at precision, SVM with information gain feature selection is a top-ranked method. These results highlight three facts: (1) XGboost is the best model when it comes to finding cell types in general (higher average accuracy). Since accuracy represents the overall correctness of the model and precision shows how good a model is at predicting a specific cell type, it is more probable to fail in finding specific cell types (less precision). More precisely, finding rare cell types with a few number of cell-specific genes is more effective in exploiting SVM and information gain. (2) Our observations confirm that XGBoost is faster and more scalable in the case of large-scale datasets, mainly because it uses its inherent feature selection simultaneously with the classification and optimization phase. (3) Compared to a tree-based model without an ensemble approach, i.e. random forest, XGBoost highlights the power of boosting strategy, either in the classification phase or feature selection phase of cell-type annotation.

In addition, an extra validation step was performed to confirm the achievements in the training phase in a more biologically meaningful scheme. The following subsections describe more details of our findings.

### 3.1. Classification Results

To explore the effect of the selected feature genes as a form of prior knowledge, we evaluated the classifiers’ performance based on the different numbers of selected features. The optimal value of features, *k*, where k=100,200,300, and 400 was determined by exploiting a greedy approach. Observing the results of the classification methods for Data1 shown in Table 2, all models reveal less misclassification rate with 400 features. In particular, SVM combined with IG gives an accuracy of 98.08%, and Precision and Recall of 87.98% and 96.76%, respectively. Additionally, *k*-NN presents a high accuracy of 96.11% when using the IG feature selection method. Moreover, random forest combined with IG delivers a high accuracy of 97.05%. XGBoost with and without IG obtains an average accuracy of 99.51% and 99.63%, respectively, which outperformed other methods. However, regarding precision, XGBoost, with only its inherent feature splitting algorithm, is the best one in the list and shows lower precision compared to the SVM with its external general-purpose feature selection method. These two methods achieved recall values with a low difference of close to zero. Additionally, the results of Data2 show almost an equivalent accuracy for SVM and XGboost methods (Table A1). *k*-NN classification method achieves high accuracy (94.66%) with 200 features selected from IG, RF, and IG combined, achieving high accuracy (96.06%) with 400 features. Lastly, SVM achieves high accuracy with 400 features (98.09%) selected from the IG feature selection method. XGBoost coupled with IG provides the best performance, with 99.67% accuracy.

For Data3, SVM outperformed the other two classification methods and achieved the highest precision of 84.91% with 300 features selected from the IG feature selection method. Regarding misclassification rate and recall, the results are very close to XGBoost. In general, Data3 has fewer features, comparatively speaking. Hence, as mentioned earlier, XGBoost is less effective when it comes to small-scale datasets.

### 3.2. Biological Validation

We performed an extra step of biological evaluation for detecting cell types using highly-ranked features identified in the feature selection phase. Among a wide range of gene set enrichment analysis (GSEA) databases, we chose the C8 collection of MSigDB, which includes cell type signature’s gene sets [18]. We separated each class’s top 20 differentially expressed genes for enrichment analysis. Table 3 shows the list of six pancreatic cell type-specific gene sets identified by the list of marker genes extracted from the feature selection phase on Data1. Additionally, as shown in Table 4, a maximum of 9 out of 20 overlapped genes between our top 20 ranked genes and pancreas gene sets were highlighted in the list. The enrichment analysis results of two other datasets are shown in Table A3 and Table A4.

## 4. Conclusions

This study compares two recently reported pioneer classification models, XGBoost and SVM, for discovering cell types using a list of marker genes. One with a blind feature selection method, pure information gain, and the other one with data sparsity-aware inherent feature selection, GXBoost feature splitting algorithm. It is shown that considering the data with its latent sparsity pattern significantly enhances the overall accuracy of the predictive models. Since the high degree of sparsity in scRNA-seq data arises from false technical zeros and true biological zeros, exploiting the patterns of existing and non-existing values for selecting biomarkers makes it more precise, faster, and more meaningful. Our study particularly demonstrates the effectiveness of ensemble tree models with an inherent sparsity-awareness feature selection approach in the cell-type automatic annotation problem. Biological validation of the results confirmed the overall accuracy of the prediction.

Moreover, the lack of canonical biomarkers for certain cell types makes it more complicated to find rare cell types using the existing genes in the list of top-ranked ones. In this case, following a manual lookup in the gene set repositories of related genes in gene sets could support the study and the results. Biologically speaking, the relation among genes is defined by structural, functional or evolutionary information. This work provides a guideline for researchers to select and apply the well-suited tool in annotating cell types using associated genes or uncovering homogeneous markers.

## Figures and Tables

**Table 1:** Details of the datasets analyzed in this study.

Dataset	Accession #	Cell Types #	Cells #	Genes #
Human Pancreatic Islets, Sample 1 (Data1)	GSM2230757	8	1937	20,125
Human Pancreatic Islets, Sample 2 (Data2)	GSM2230758	8	1724	20,125
Mouse Pancreatic Islets, Sample 2 (Data3)	GSM2230762	8	1064	14,878

**Table 2:** Comparison of classification results for Data1.

Method	No of Features	Accuracy %	Precision %	Recall %
SVM + IG	400	98.08	87.98	**96.76 **
RF + IG	400	97.05	77.48	96.52
*k*NN + IG	400	96.11	77.53	96.51
XGBoost + IG	400	**99.51**	80.45	91.68
**XGBoost**	400	**99.63**	**88.41**	**96.38**

**Table 3:** List of eight gene sets correlated to the Pancreatic cell types of Data1 resulting from the GSEA analysis.

Pancreas Gene Set Name	Dataset
Muraro pancreas endothelial cell [362]	Data1, Data2, Data3
Muraro pancreas mesenchymal stromal ce cell [681]	Data1, Data2, Data3
Muraro pancreas acinar cell [732]	Data1, Data2, Data3
Muraro pancreas ductal cell [1276]	Data1, Data2, Data3
Muraro pancreas alpha cell [568]	Data1, Data2
Descartes fetal pancreas islet endocricrine cells [170]	Data1, Data2
Muraro Pancreas Epsilon Cell [44]	Data2
Muraro Pancreas Delta Cell [250]	Data2

**Table 4:** List of 9 out of 20 overlapped genes between our top 20 ranked genes and pancreas gene sets (Data1).

Gene Symbol	Description of Functionality
IFITM3	interferon induced transmembrane protein 3 [Source:HGNC Symbol;Acc:HGNC:5414]
IGFBP4	insulin like growth factor binding protein 4 [Source:HGNC Symbol;Acc:HGNC:5473]
IFITM2	interferon induced transmembrane protein 2 [Source:HGNC Symbol;Acc:HGNC:5413]
COL4A1	collagen type IV alpha 1 chain [Source:HGNC Symbol;Acc:HGNC:2202]
SPARC	secreted protein acidic and cysteine rich [Source:HGNC Symbol;Acc:HGNC:11219]
IGFBP7	insulin like growth factor binding protein 7 [Source:HGNC Symbol;Acc:HGNC:5476]
VIM	vimentin [Source:HGNC Symbol;Acc:HGNC:12692]
TM4SF1	transmembrane 4 L six family member 1 [Source:HGNC Symbol;Acc:HGNC:11853]
HLA-B	“major histocompatibility complex, class I, B [Source:HGNC Symbol;Acc:HGNC:4932]”

## Data Availability

The Pancreas scRNA-seq datasets presented in this study are openly available in NCBI GEO Datasets at https://www.ncbi.nlm.nih.gov/geo/query/acc.cgi?acc=GSE84133 accessed on 1 January 2020, and the code is publicly available at Github https://github.com/yashcoder007/Supervised-Cell-Type-Identification-MDPI---Genes, accessed on 1 January 2020.

## Appendix A

**Table A1:** Comparison of classification results for Data2.

Method	No of Features	Accuracy %	Precision %	Recall %
SVM + IG	400	98.09	**79.04 **	98.08
RF + IG	400	96.06	65.86	94.76
*k*NN + IG	200	94.66	65.68	96.63
**XGBoost + IG**	400	**99.67**	75.63	96.62
**XGBoost**	400	**99.78**	76.83	**99.22**

**Table A2:** Comparison of classification results for Data3.

Method	No of Features	Accuracy %	Precision %	Recall %
SVM + IG	300	**99.23 **	**91.95**	**93.29**
RF + IG	400	99.03	67.26	88.68
*k*NN + IG	400	98.60	73.63	88.01
XGBoost + IG	400	**99.42**	80.72	90.58
**XGBoost**	400	**99.18**	78.91	**93.17**

**Table A3:** List of 17 out of 20 overlapped genes between our top 20 ranked genes and pancreas gene sets (Data2).

Gene Symbol	Description of Functionality
PMEPA1	prostate transmembrane protein, androgen induced 1 [Source:HGNC Symbol;Acc:HGNC:14107]
TACSTD2	tumor associated calcium signal transducer 2 [Source:HGNC Symbol;Acc:HGNC:11530]
KRT7	keratin 7 [Source:HGNC Symbol;Acc:HGNC:6445]
SDC4	syndecan 4 [Source:HGNC Symbol;Acc:HGNC:10661]
SOX4	SRY-box transcription factor 4 [Source:HGNC Symbol;Acc:HGNC:11200]
KRT19	keratin 19 [Source:HGNC Symbol;Acc:HGNC:6436]
FLNA	filamin A [Source:HGNC Symbol;Acc:HGNC:3754]
FXYD3	FXYD domain containing ion transport regulator 3 [Source:HGNC Symbol;Acc:HGNC:4027]
IFITM3	interferon induced transmembrane protein 3 [Source:HGNC Symbol;Acc:HGNC:5414]
SERPING1	serpin family G member 1 [Source:HGNC Symbol;Acc:HGNC:1228]
COL18A1	collagen type XVIII alpha 1 chain [Source:HGNC Symbol;Acc:HGNC:2195]
PCSK1	proprotein convertase subtilisin/kexin type 1 [Source:HGNC Symbol;Acc:HGNC:8743]
HADH	hydroxyacyl-CoA dehydrogenase [Source:HGNC Symbol;Acc:HGNC:4799]
MAFA	MAF bZIP transcription factor A [Source:HGNC Symbol;Acc:HGNC:23145]
ARX	aristaless related homeobox [Source:HGNC Symbol;Acc:HGNC:18060]
IRX2	iroquois homeobox 2 [Source:HGNC Symbol;Acc:HGNC:14359]
GC	GC vitamin D binding protein [Source:HGNC Symbol;Acc:HGNC:4187]

**Table A4:** List of 15 out of 20 overlapped genes between our top 20 ranked genes and pancreas gene sets (Data3).

Gene Symbol	Description of Functionality
SPARC	secreted protein acidic and cysteine rich [Source:HGNC Symbol;Acc:HGNC:11219]
COL4A1	collagen type IV alpha 1 chain [Source:HGNC Symbol;Acc:HGNC:2202]
FLT1	fms related receptor tyrosine kinase 1 [Source:HGNC Symbol;Acc:HGNC:3763]
PECAM1	platelet and endothelial cell adhesion molecule 1 [Source:HGNC Symbol;Acc:HGNC:8823]
SERPINH1	serpin family H member 1 [Source:HGNC Symbol;Acc:HGNC:1546]
COL4A2	collagen type IV alpha 2 chain [Source:HGNC Symbol;Acc:HGNC:2203]
IGFBP7	insulin like growth factor binding protein 7 [Source:HGNC Symbol;Acc:HGNC:5476]
CDH5	cadherin 5 [Source:HGNC Symbol;Acc:HGNC:1764]
VIM	vimentin [Source:HGNC Symbol;Acc:HGNC:12692]
PMEPA1	prostate transmembrane protein, androgen induced 1 [Source:HGNC Symbol;Acc:HGNC:14107]
MSN	moesin [Source:HGNC Symbol;Acc:HGNC:7373]
S100A16	S100 calcium binding protein A16 [Source:HGNC Symbol;Acc:HGNC:20441]
ANXA2	annexin A2 [Source:HGNC Symbol;Acc:HGNC:537]
CD24	CD24 molecule [Source:HGNC Symbol;Acc:HGNC:1645]
NFIB	nuclear factor I B [Source:HGNC Symbol;Acc:HGNC:7785]
